# Prognostic factors for employment outcomes in patients with a history of childhood-onset drug-resistant epilepsy

**DOI:** 10.3389/fped.2023.1173126

**Published:** 2023-07-28

**Authors:** Yuto Arai, Tohru Okanishi, Hisashi Noma, Sotaro Kanai, Tatsuya Kawaguchi, Hiroshi Sunada, Ayataka Fujimoto, Yoshihiro Maegaki

**Affiliations:** ^1^ Division of Child Neurology, Department of Brain and Neurosciences, Faculty of Medicine, Tottori University, Yonago, Japan; ^2^ Department of Data Science, The Institute of Statistical Mathematics, Tokyo, Japan; ^3^ Advanced Medicine, Innovation and Clinical Research Center, Tottori University Hospital, Yonago, Japan; ^4^ Comprehensive Epilepsy Center, Seirei Hamamatsu General Hospital, Shizuoka, Japan

**Keywords:** drug-resistant epilepsy, childhood-onset epilepsy, risk factors, non-employment, psychiatric disorders, special needs education

## Abstract

**Objective:**

The employment outcomes of childhood-onset drug-resistant epilepsy (DRE) has not been studied enough. The aim of this retrospective cohort study is to investigate the employment outcomes of childhood-onset DRE in June 2022 and identify the risk factors associated with non-employment.

**Materials and methods:**

The sample consisted of 65 participants ≥18 years of age with a history of childhood-onset DRE. Fifty participants (77%) were salaried employees and 15 participants (23%) were non-employed. Clinical and psychosocial information were evaluated for calculating the relative risk (RR) of non-employment.

**Results:**

Regarding medical factors, lower IQ [RR, 0.645; 95% confidence interval (CI), 0.443–0.938; *p *= 0.022] was positively associated with employment. In contrast, age at follow-up (RR, 1.046; 95% CI, 1.009–1.085; *p *= 0.014); number of ASMs at follow-up (RR, 1.517; 95% CI, 1.081–2.129; *p *= 0.016); use of medications such as phenobarbital (RR, 3.111; 95% CI, 1.383–6.997; *p *= 0.006), levetiracetam (RR, 2.471; 95% CI, 1.056–5.782; *p *= 0.037), and topiramate (RR, 3.576; 95% CI, 1.644–7.780; *p *= 0.001) were negatively associated with employment. Regarding psychosocial factor, initial workplace at employment support facilities (RR, 0.241; 95% CI, 0.113–0.513; *p* < 0.001) was positively associated with employment. In contrast, complication of psychiatric disorder symptoms (RR, 6.833; 95% CI, 2.141–21.810; *p* = 0.001) was negatively associated with employment. Regarding educational factor, graduating schools of special needs education (RR, 0.148; 95% CI, 0.061–0.360; *p* < 0.001) was positively associated with employment.

**Conclusions:**

Specific medical, psychosocial, and educational factors may influence the employment outcomes of childhood-onset DRE. Paying attention to ASMs’ side effects, adequately preventing the complications of psychiatric disorder symptoms, and providing an environment suitable for each patient condition would promote a fine working status for people with childhood-onset DRE.

## 1. Introduction

Epilepsy is a prevalent neurological disorder associated with numerous comorbidities that adversely influence one’s quality of life, education, employment, and social adjustment (1). The prevalence of epilepsy in the general population is estimated to be 0.5% (2). Understanding the natural history of childhood-onset epilepsy is imperative for improving the management and providing children, parents, and families with prognostic information (3). Approximately 50% of patients with newly diagnosed epilepsy become seizure-free with the initially prescribed anti-seizure medications (ASMs). Despite the approval of new ASMs, 30% of patients continue to have seizures after treatment with at least two different ASMs, either successively or concomitantly (4–7).

The social outcomes of adults with a history of childhood-onset epilepsy are often unsatisfactory (8, 9), and the reasons for poor social outcomes in adulthood are poorly understood (9). Several population-based studies concluded that maintaining normal intelligence and early seizure control are important for favorable long-term social outcomes in patients with childhood-onset epilepsy (3, 10, 11). However, in patients of intractable seizures, recurrent seizures seriously affect the development of the nervous system, resulting in growth retardation, low cognitive function, and a series of neuropsychological problems; their social outcomes are poor in all aspects (3, 12–15). Therefore, there is a need to develop a strategy to intervene in this vulnerable group (9).

Employment is one of the factors that determine social outcomes and has a major contribution to the quality of life of people with epilepsy (16). Having a job not only supports financial independence but also consolidates self-esteem and promotes social functioning (17). Drug-resistant epilepsy (DRE) is associated with non-employment (18); however, the risk factors related to non-employment are not well understood. The purpose of this study is to investigate the employment outcomes of people with a history of childhood DRE and identify the risk factors related to non-employment.

## 2. Methods

### 2.1. Participants

We retrospectively reviewed the medical records of participants with childhood-onset DRE who visited the Tottori University Hospital between 1969 and 2022. DRE is defined by the International League Against Epilepsy as the persistence of seizures despite the use of at least two adapted ASMs at efficacious daily doses (19).

The inclusion criteria were as follows: (i) participants diagnosed with DRE at less than 16 years of age and (ii) participants older than 18 years at the final follow-up in June 2022. Therefore, even though participants are not currently having DRE, if they met the definition of DRE in childhood, they were included in the study. The exclusion criteria were as follows: (i) participants with severe motor and intellectual disabilities as per Oshima’s classification (20) and (ii) participants whose employment status in June 2022 was unavailable due to transition or hospital transfer.

### 2.2. Employment status

We collected data on the employment status in June 2022 from the most recent medical records or official documents and divided them into two groups: an employment group and a non-employment group. We defined the employment group as the salaried employee group working in a regular workplace, including a typical company, industry, farm, or disability employment service, as of June 2022. Disability employment services are facilities for securing workplaces for persons with disabilities and improving their knowledge and abilities, based on the Services and Supports for Persons with Disabilities Act. The details of the service vary depending on the degree of disability; however, a minimum wage is guaranteed. Further, we defined the non-employment group as a group that has never been employed, has left services, or has been terminated.

### 2.3. Clinical characteristics

#### 2.3.1. Medical characteristics

We collected information on age at follow-up, sex, number of ASMs at follow-up, onset age of epilepsy, Magnetic resonance imaging (MRI) findings, intellectual functioning, seizure frequency during the last year, type of epilepsy, epilepsy syndrome, and etiology of epilepsy. Regarding intellectual functioning, intellectual disability (ID) was defined as an intelligence quotient (IQ) score below 70. The IQ scores were divided into five categories: mild, moderate, severe, and profound ID, with scores of 51–70, 36–50, 21–35, and <20, respectively (21). For participants without IQ scores, the word description (profound, severe, moderate, etc.) in the medical record was considered. Seizure frequencies were categorized as yearly (1–11 seizures/year), monthly (1–3 seizures/month), weekly (1–6 seizures/week), and daily (more than one seizure/day) (22). Epilepsy was categorized according to recent recommendations (23).

#### 2.3.2. Psychosocial characteristics

We collected psychosocial information, including psychiatric and neurodevelopmental disorder symptoms, living environment (facility or home), and place of residence (inside or outside Tottori Prefecture). Psychiatric disorder symptoms include conduct disorder (keyword searching of “violence”, “rant”, “aggressive”, “crime” or “misconduct”), mood disorder (keyword searching of “depression” or “manic”), schizophrenic disorder (key word searching of “hallucination” or “delusion”), and post-traumatic stress disorder (PTSD) symptoms (keyword searching of “psychological trauma”). Neurodevelopmental disorder symptoms include attention deficit hyperactivity disorder (ADHD) (keyword searching of “hyperactive” or “impulsive,”) and autism spectrum disorder (ASD) symptoms (keyword searching of “sticking” or “autistic”).

#### 2.3.3. Educational characteristics

We collected educational information, including type of schools, history of school refusal, and years from education completion. Regarding the type of schools, we divided the schools that the participants attended prior to employment into two categories: regular schools and schools for special needs education. Regular schools include elementary, junior high, and high schools. In the Japanese education system, compulsory education is provided to children aged 6–15 years [(elementary school (6–12 years) and junior high school (12–15 years)]. After completing compulsory education, 98.5% of children in the Tottori prefecture pass an entrance examination for high school (15–18 years), after which they continue their schooling for 3 years (24). In contrast, schools for special needs education are defined as schools for children with severe disabilities. In schools for special needs education, children learn through a special curriculum while being supported by special education teachers who use various facilities and equipment to meet the needs of those children. These schools comprise four levels of education: kindergarten (4–6 years), elementary (6–12 years), lower secondary (12–15 years), and upper secondary (15–18 years) (25).

### 2.4. Statistical analysis

We performed the modified Poisson regression analyses (26) to assess the associations between non-employment and the potential prognostic factors. Missing data were treated by the multiple imputation by chained equation with 200 imputations (27). A two-tailed *p*-value of less than 0.05 was considered statistically significant. Association with employment was defined as positive if the relative risk (RR) of non-employment showed <1 (*p *< 0.05) and defined as negative if RR of non-employment showed >1 (*p *< 0.05). Multivariate analysis was avoided due to the limited number of cases. Data analysis was performed using R ver. 4.2.1 (R Foundation for Statistical Computing, Vienna, Austria).

### 2.5. Ethical compliance

This study was approved by the Institutional Ethics Committee of the Tottori University Hospital (approval number: 22A092). Informed consent was obtained from all participants using the opt-out condition on a website [https://www2.hosp.med.tottori-u.ac.jp/departments/center/amirt/2115/3186/3294/21841.html (in Japanese)].

## 3. Results

A total of 91 participants were initially included in the study. Among these, 8 participants met exclusion criteria (i) and 18 participants met exclusion criteria (ii). Finally, 65 participants were included in the study.

Of the 65 participants, 50 participants (76.9%) were employed, and 15 (23.1%) were non-employed. 47 (94.0%) of the employed participants worked in a disability employment service, and the rest 3 (6.0%) worked in a regular workplace. 12 (80.0%) of the non-employed participants had left services or were terminated, and the rest 3 (20.0%) had never been employed.

### 3.1. Clinical characteristics

#### 3.1.1. Medical characteristics

The medical characteristics are presented in Table 1. The age at follow-up was 18–53 years (mean age: 31 years). More than 90% of the participants had an IQ of less than 70. Epilepsy was classified as focal epilepsy in 45 participants (69.2%), generalized epilepsy in 4 (6.2%), and combined generalized and focal epilepsy in 11 (16.9%). Epilepsy syndromes were identified in 17 participants (26.2%): temporal lobe epilepsy in two (3.1%), atypical benign partial epilepsy in childhood in one (1.5%), Lennox–Gastaut syndrome in two (3.1%), infantile epileptic spasms syndrome in six (9.2%), Dravet syndrome in five (7.7%), and early infantile epileptic encephalopathy with burst-suppression in one (1.5%). The etiologies of epilepsy were identified in 23 participants (35.4%): structural in 13 (20.0%), genetic in 9 (13.8%), and metabolic in 1 (1.5%).

**Table 1:** Medical characteristics of the participants.

Characteristic	All cases *N* = 65	Employed group *N* = 50	Non-employed group *N* = 15	*p*-value
Gender				
Male	37 (56.9%)	30 (60.0%)	7 (47.0%)	0.39
Onset age of epilepsy, means (range) *N* = 64	3.78 (0–15)	3.345 (0–15)	5.2 (0–12)	0.1
Age at follow up, mean (range)	31 (18–56)	29.82 (18–53)	34.867 (24–56)	0.04
Number of ASMs at follow up, mean (range)	2.4 (1–5)	2.24 (1–5)	3 (1–5)	0.03
1	15 (23.1%)	13 (26.0%)	2 (13.3%)	
2	22 (33.8%)	19 (38.0%)	3 (20.0%)	
3	18 (27.7%)	13 (26.0%)	5 (33.3%)	
4	6 (9.2%)	3 (6.0%)	3 (20.0%)	
5	4 (6.2%)	2 (4.0%)	2 (13.3%)	
Intelligence quotient				
>70	2 (3.1%)	1 (2.1%)	1 (6.7%)	0.38
51–70	17 (26.2%)	11 (22.9%)	6 (40.0%)	0.16
36–50	5 (7.7%)	3 (6.3%)	2 (13.3%)	0.29
21–35	18 (27.7%)	16 (33.3%)	2 (13.3%)	0.31
20>	19 (29.2%)	17 (35.4%)	2 (13.3%)	0.31
Unknown	4 (6.2%)	2 (4.0%)	2 (13.3%)	
Seizure frequency				
Daily	4 (6.2%)	4 (8.0%)	0 (0%)	0.57
Weekly	17 (26.2%)	13 (26.0%)	4 (26.7%)	1
Monthly	9 (13.8%)	5 (10.0%)	4 (26.7%)	0.2
Yearly	9 (13.8%)	6 (12.0%)	3 (20.0%)	0.43
Seizure free over 1 year	25 (38.5%)	21 (42.0%)	4 (26.7%)	0.37
Unknown	1 (1.5%)	1 (2.0%)	0 (0%)	
Type of epilepsy				
Focal	44 (67.7%)	32 (64.0%)	12 (80.0%)	0.35
Generalized	5 (7.7%)	5 (10.0%)	0 (0%)	0.58
Combined	10 (15.4%)	8 (16.0%)	2 (13.3%)	1
Unknown	6 (9.2%)	5 (10.0%)	1 (6.7%)	1
Etiology				
Structual	6 (9.2%)	4 (8.0%)	2 (13.3%)	0.62
Genetic	8 (12.3%)	6 (12.0%)	2 (13.3%)	1
Metabolic	2 (3.1%)	1 (2.0%)	1 (6.7%)	0.41
Infectious	6 (9.2%)	6 (12.0%)	0 (0.0%)	0.32
Immune	1 (0.0%)	0 (0.0%)	1 (6.7%)	0.07
Unknown	42 (64.6%)	33 (66.0%)	9 (60%)	0.76

ASMs, antiseizure medications.

#### 3.1.2. Psychosocial characteristics

The psychosocial information are shown in Table 2. Regarding psychiatric disorder symptoms, 15 participants (23.1%) had conduct disorder symptoms, 6 (9.2%) had mood disorder symptoms, 3 (4.6%) had schizophrenic disorder symptoms, and 5 (7.7%) had PTSD symptoms. Any of these four psychiatric disorder symptoms was recognized in 24 participants (36.9%). Regarding neurodevelopmental disorder symptoms, 16 participants (24.6%) had ADHD symptoms and 22 (33.8%) had ASD symptoms. Any of these two neurodevelopmental disorder symptoms were recognized in 30 participants (46.2%). 55 participants (84.6%) lived at home and 52 (80.0%) lived inside Tottori prefecture.

**Table 2:** Psychosocial and educational characteristics of the participants.

	All cases *N* = 65	Employed group *N* = 50	Non-employed group *N* = 15	*p*-value
Psychiatric symptoms	24 (36.9%)	12 (24.0%)	12 (80.0%)	<0.001
Conduct disorder symptoms	15 (23.1%)	9 (18.0%)	6 (40.0%)	0.09
Mood disorder symptoms	6 (9.2%)	1 (2.0%)	5 (33.3%)	0.002
Schizophrenic disorder symptoms	3 (4.6%)	0 (0.0%)	3 (20.0%)	0.01
PTSD symptoms	5 (7.7%)	3 (6.0%)	2 (13.3%)	0.33
Neurodevelopmental disorder symptoms	30 (46.2%)	23 (46.0%)	7 (46.7%)	
ADHD symptoms	16 (24.6%)	12 (24.0%)	4 (26.7%)	1
ASD symptoms	22 (33.8%)	16 (32.0%)	6 (40.0%)	0.76
Education environment				
Graduating from regular high school	12 (18.5%)	3 (6.0%)	9 (60.0%)	<0.001
Graduating from school for special needs education (upper secondary department)	45 (69.2%)	40 (80.0%)	5 (33.3%)	
Unknown	8 (12.3%)	7 (14.0%)	1 (6.7%)	
History of school refusal				
Yes	5 (7.7%)	4 (8.0%)	1 (6.7%)	1
No	54 (83.1%)	40 (80.0%)	14 (93.3%)	
Unknown	6 (9.2%)	6 (12.0%)	0 (0%)	
Years from education completion, mean (range)	11.91 (0–38)	10.23 (0–27)	17.07 (6–38)	0.008
Living arrangements				
Home	55 (84.6%)	44 (88.0%)	11 (73.3%)	0.22
Supported accomodation	10 (15.4%)	6 (12.0%)	4 (26.7%)	
Living place				
Inside Tottori Prefecture	52 (80.0%)	40 (80.0%)	12 (80.0%)	1
Outside Tottori Prefecture	13 (20.0%)	10 (20.0%)	3 (20.0%)	
Initial workplace				
Disability employment services	55 (84.6%)	47 (94%)	8 (53.3%)	0.003
Regular workplace	7 (10.8%)	3 (6.0%)	4 (26.7%)	0.004
None	3 (4.6%)	0 (0.0%)	3 (20.0%)	

PTSD, post-traumatic stress disorder; ADHD, attention deficit hyperactivity disorder; ASD, autism spectrum disorder.

#### 3.1.3. Educational characteristics

The educational characteristics are shown in Table 2. Regarding education, 12 participants (18.5%) had graduated from regular schools and 45 (69.2%) had completed special needs education. Years from education completion was 0–38 years (mean years: 11.91 years).

### 3.2. Association between clinical characteristics and employment

#### 3.2.1. Factors associated with medical characteristics

Older age at follow-up [RR, 1.046; 95% confidence interval (CI), 1.009–1.085; *p *= 0.014] and a greater number of antiepileptic drugs taken at follow-up (RR, 1.517; 95% CI, 1.081–2.129; *p *= 0.016) were negatively associated with employment (Table 3). Regarding ASMs, use of phenobarbital (PB) (RR, 3.111; 95% CI, 1.383–6.997; *p *= 0.006), levetiracetam (LEV) (RR, 2.471; 95% CI, 1.056–5.782; *p *= 0.037), and topiramate (TPM) (RR, 3.576; 95% CI, 1.644–7.780; *p* = 0.001) were negatively associated with employment (Table 4). In contrast, lower IQ was positively associated with employment (RR, 0.645; 95% CI, 0.443–0.938; *p* = 0.022). Seizure frequency, abnormalities on MRI, and onset age of epilepsy were not significantly associated with employment.

**Table 3:** Univariate analyses of RR for non-employment.

	RR	95% CL	95% CU	*p*-value
Male (*N* = 65)	1.510	0.622	3.669	0.363
Age at follow up (means) (*N* = 65)	1.046	1.009	1.085	0.014
Onset age of epilepsy (mean) (*N* = 64)	1.069	0.986	1.159	0.107
Number of ASMs at follow up (mean) (*N* = 65)	1.517	1.081	2.129	0.016
Abnormalities on MRI (*N* = 49)	1.195	0.431	3.316	0.732
Intelligence Quotient (*N* = 61)	0.645	0.443	0.938	0.022
Seizure frequency (*N* = 64)	1.034	0.782	1.366	0.816
Psychiatric symptoms (*N* = 65)	6.833	2.141	21.81	0.001
Conduct disorder symptoms	2.222	0.943	5.235	0.068
Mood disorder symptoms	4.917	2.519	9.595	<0.001
Schizophrenic symptoms	5.167	3.108	8.588	<0.001
PTSD symptoms	1.846	0.569	5.986	0.307
Neurodevelopmental disorder symptoms (*N* = 65)				
ADHD symptoms	1.114	0.412	3.014	0.832
ASD symptoms	1.303	0.532	3.193	0.563
Education environment (*N* = 57)				
Graduating from school for special needs education	0.148	0.061	0.360	<0.001
History of school refusal (*N* = 59)	0.771	0.126	4.714	0.779
Living arrangements (*N* = 65)				
Home	0.500	0.198	1.261	0.142
Living place (*N* = 65)				
Inside tottori Prefecture	1.000	0.330	3.033	1.000
Initial workplace (*N* = 62)				
Disability employment services	0.241	0.113	0.513	<0.001
Regular workplace	3.013	1.309	6.933	0.009

RR, relative risk; 95% CL, confidence interval lower limits; 95% CU, confidence interval upper limits; ASMs, antiseizure medications; PTSD, post-traumatic stress disorder; ADHD, attention deficit hyperactivity disorder; ASD, autism spectrum disorder.

**Table 4:** ASMs in employment and non-employment groups.

	Employment group *N* = 50 *N* (%)	Non-employment group *N* = 15 *N* (%)	RR (95% CI)	*p*-value
Phenobarbital	4 (8.0)	5 (33.3)	3.112 (1.383–6.997)	0.006
Valproate	17 (34.0)	6 (40.0)	1.218 (0.495–2.992)	0.668
Carbamazepine	18 (36.0)	5 (33.3)	0.914 (0.355–2.350)	0.850
Levetiracetam	10 (20.0)	7 (46.7)	2.471 (1.056–5.782)	0.037
Lacosamide	7 (14.0)	2 (13.3)	0.958 (0.258–3.554)	0.948
Lamotrigine	12 (24.0)	6 (40.0)	1.741 (0.723–4.191)	0.216
Perampanel	5 (10.0)	1 (6.7)	0.703 (0.111–4.453)	0.708
Topiramate	2 (4.0)	4 (26.7)	3.576 (1.644–7.780)	0.001
Clonazepam	4 (8.0)	1 (6.7)	0.858 (0.140–5.248)	0.868
Zonisamide	6 (12.0)	1 (6.7)	0.592 (0.091–3.844)	0.583
Phenytoin	3 (6.0)	3 (20.0)	2.459 (0.954–6.332)	0.062
Clobazam	12 (24.0)	1 (6.7)	0.286 (0.041–1.979)	0.205
Gabapentin	1 (2.0)	1 (6.7)	NA	
Acetazolamide	1 (2.0)	1 (6.7)	NA	
Benzodiazepine	1 (2.0)	0 (0)	NA	
Sultiame	1 (2.0)	0 (0)	NA	
Stiripentol	4 (8.0)	0 (0)	NA	
Potassium bromide	1 (2.0)	0 (0)	NA	
Rufinamide	1 (2.0)	0 (0)	NA	

ASMs, antiseizure medications; RR, relative risk; CI, confidential interval; NA, not applicable for statistics because of the small numbers.

#### 3.2.2. Factors associated with psychosocial characteristics

Complications of mood (RR, 4.917; 95% CI, 2.519–9.595; *p *< 0.001), schizophrenic disorder symptoms (RR, 5.167; 95% CI, 3.108–8.588; *p *< 0.001), and any of the four psychiatric disorder symptoms (RR, 6.833; 95% CI, 2.141–21.810; *p *= 0.001) were negatively associated with employment. In contrast, complications of neurodevelopmental disorder (ADHD and ASD) symptoms were not significantly associated with employment status.

Initial workplaces at employment support facilities (RR, 0.241; 95% CI, 0.113–0.513; *p *< 0.001) were positively associated with employment. In contrast, initial workplaces at regular companies (RR, 3.013; 95% CI, 1.309–6.933; *p *= 0.009) were negatively associated with employment. Living environment and place of residence were not associated with employment.

#### 3.2.3. Factors associated with educational characteristics

Graduating schools for special needs education (RR, 0.148; 95% CI, 0.061–0.360; *p *< 0.001) was positively associated with employment. History of school refusal was not significantly associated with employment.

## 4. Discussion

The present study comprised a long-term follow-up evaluation of the employment outcomes of 65 participants aged 18 years or older who had a history of childhood-onset DRE. Most participants with childhood-onset DRE were able to work as salaried employees with social support.

In contrast, approximately one of every five participants was non-employed. There were significant risk factors for non-employment in terms of both medical and social aspects.

### 4.1. Factors associated with medical characteristics

Use of PB, LEV, and TPM were negatively associated with employment status. PB causes drowsiness, irritation, depression, mental illness, hallucination (28), LEV causes somnolence, asthenia, dizziness, and behavioral difficulties (29); and TPM causes fatigue, cognitive dysfunction, drowsiness, and diminished psychomotor movement (30). Therefore, these ASMs may have caused cognitive or psychological problems that made it difficult for the participants to continue working. The ultimate goal of ASM therapy is to restore normal health-related quality of life without causing any clinically significant adverse drug effects (31). ASMs that may cause psychosocial problems should be avoided from the perspective of employment.

A lower IQ was positively associated with employment although people with ID are three to four times less likely to have a job than people without ID (32). Verdonschot stated that environmental factors such as emotional support, positive staff attitudes, staff-initiated interactions, and more staff attention positively influence social participation among people with ID (33). Most of the patients were working at disability employment services and were thought to have received staff support; therefore, the severity of IQ may not have mattered significantly (Supplementary Figure S1).

Seizure frequency was not significantly associated with non-employment, although frequent seizures, especially during work, may make it infeasible to keep epilepsy diagnosis private, thus undermining the chance of sustaining employment (18). We presumed that the frequency of seizures may not matter much for employment, probably because many participants were employed at disability employment services and the surrounding people may have an understanding of epilepsy.

### 4.2. Factors associated with psychosocial characteristics

Comorbidity of depression, schizophrenia, and any of the four psychiatric symptom was negatively associated with employment. Patients with epilepsy have two- to three-fold more complications of psychiatric disorder symptoms, such as mood disorders, anxiety, and psychotic disorders, than the general population, and approximately a third of patients with intractable epilepsy are affected by these symptoms (34–36). Depression liability appears to increase non-employment, particularly by increasing disability (37). The employment rate among people with schizophrenia is only 10%–30% (38). Therefore, it may be important to adequately manage the comorbidity of psychiatric symptoms, especially those of depression and schizophrenia.

Initial workplace at disability employment services was positively associated with employment. A better understanding of the effects of employment characteristics is essential, and interventions on employment characteristics could reduce the impact of disability and structural inequalities on mental health (39–41). Patients with epilepsy tend to be stigmatized, and this tendency has been stronger in unemployed patients (18, 42). Therefore, it may be important for these patients to work in an environment where epilepsy is less likely to be stigmatized. In this respect, disability employment services may reduce mental burden more than regular companies.

### 4.3. Factors associated with educational characteristics

Graduating from schools for special needs education was positively associated with employment. In-school services are critical in predicting current employment for individuals with ID, and any individual with mild, moderate, or severe ID could benefit from in-school services to prepare for future employment (43). The policy of the Japanese government is to guarantee special needs education to meet the individual needs of each child, regardless of the nature or severity of the disability, in all areas of the country (44). The government also provides carefully planned education to maximally develop the children’s capabilities and cultivate their ability to participate independently in society. Therefore, handicapped children will eventually have a better social prognosis if they receive adequate educational support.

### 4.4. Limitations

Our study has several limitations. Firstly, we did not investigate the genetic factors. Genetic polymorphisms are associated with drug-specific treatment efficacy, unwanted drug reactions, and the long-term prognosis of patients with epilepsy (45). Therefore, managing these issues can lead to more favorable clinical conditions in childhood, promote good adaptation and also employment outcomes in adulthood. Secondly, there might be selection bias. We selected participants with the most severe childhood-onset DRE because they need to visit advanced medical institutions, such as university hospitals, instead of local clinics, and have difficulty transitioning to an adult neurology department due to medical or social severity. Thirdly, we did not investigate details of cognitive functions in this study although employment status is closely related to cognitive functioning (46). People with epilepsy are predisposed to social cognitive deficits owing to pathophysiological, psychiatric, or psychosocial problems (47). Fourthly, we did not investigate the resolution of psychiatric symptoms reduces the risk of non-employment.

Therefore, we should conduct another future study to investigate whether appropriate management of genetic polymorphisms and psychiatric disorders and evaluation of cognitive function will lead to employment promotion.

## 5. Conclusions

Previous reports investigating the social outcomes of childhood-onset epilepsy insisted that the presence of ID and poor seizure control were significant risk factors for poor employment prognosis (3, 10, 11). However, our study, which investigated patients with childhood-onset DRE, has yielded conflicting results. These results appear to be due to the fact that patients with DRE were more likely to work at disability employment services, which minimized the negative impact of seizure frequency and intellectual level on employment.

Our study also suggests that psychiatric symptoms may affect employment prognosis. Specific ASMs that affect cognition and mental health may also be related to non-employment. Therefore, it was thought that paying attention to ASMs’ side effects, adequately managing the complications of psychiatric disorder symptoms, and providing an environment suitable for employment would promote a fine working status for people with childhood-onset DRE. However, larger population-based studies are warranted to validate these risk factors.

## Data Availability

The raw data supporting the conclusions of this article will be made available by the authors, without undue reservation.
