# Supplementary figures and images for: Prognostic factors for employment outcomes in patients with a history of childhood-onset drug-resistant epilepsy
Source: Front Pediatr. 2023 Jul 28;11:1173126. doi: 10.3389/fped.2023.1173126 (PMC10419209; doi:10.3389/fped.2023.1173126)

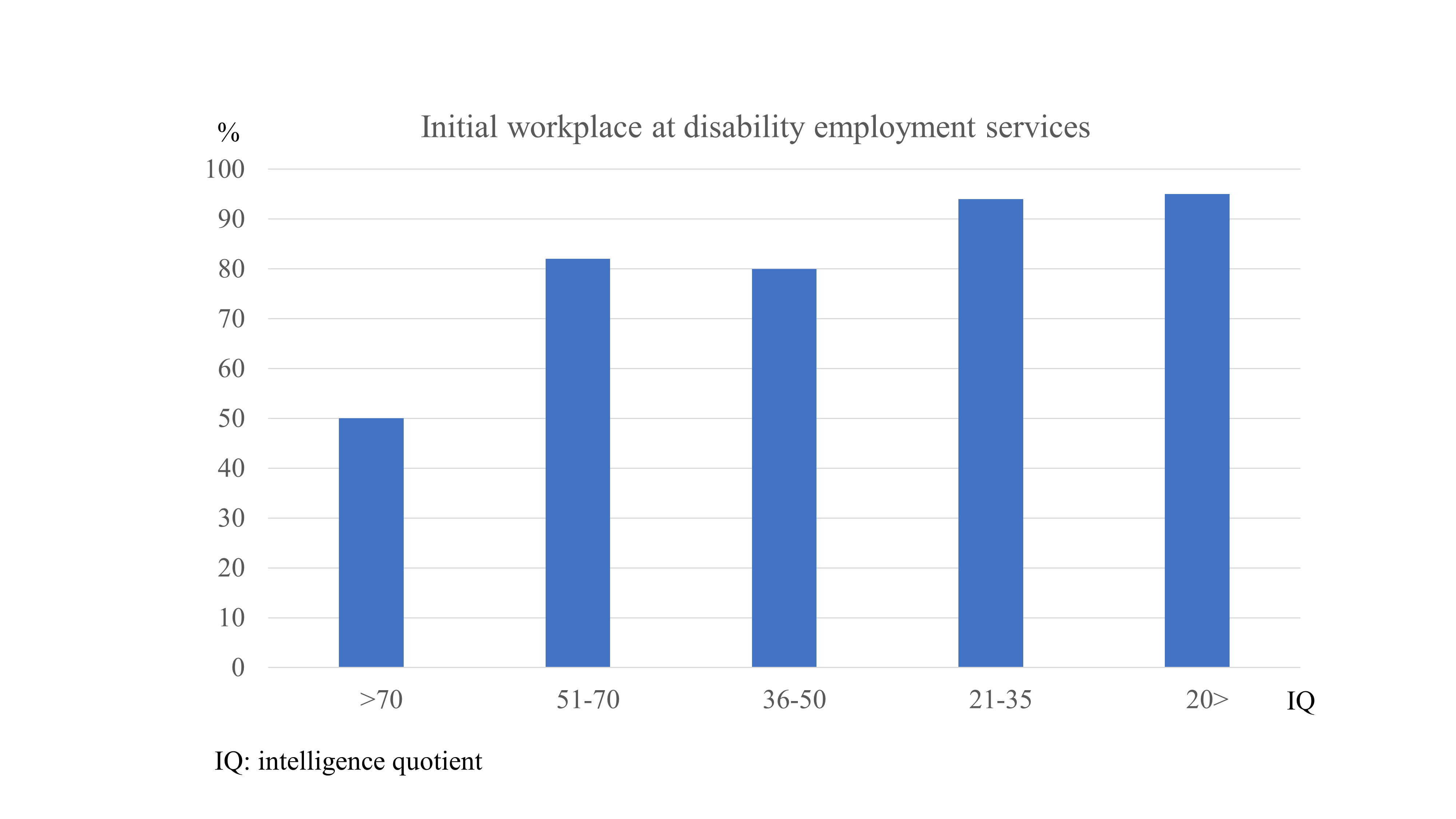

Supplement: Supplementary file 1 [file Image1.tif]
